# Neuron-glia networks: integral gear of brain function

**DOI:** 10.3389/fncel.2014.00378

**Published:** 2014-11-06

**Authors:** Gertrudis Perea, Mriganka Sur, Alfonso Araque

**Affiliations:** ^1^Functional and System Neurobiology, Instituto Cajal, Consejo Superior de Investigaciones CientíficasMadrid, Spain; ^2^Department of Brain and Cognitive Sciences, Picower Institute for Learning and Memory, Massachusetts Institute of TechnologyCambridge, MA, USA; ^3^Department of Neuroscience, University of Minnesota, MinneapolisMN, USA

**Keywords:** astrocytes, neuron-glia network, synaptic plasticity, gliotransmission, information coding

## Abstract

Astrocytes, the most abundant glial cell in the brain, play critical roles in metabolic and homeostatic functions of the Nervous System; however, their participation in coding information and cognitive processes has been largely ignored. The strategic position of astrocyte processes facing synapses and the astrocyte ability to uptake neurotransmitters and release neuroactive substances, so-called “gliotransmitters”, provide the scenario for prolific neuron-astrocyte signaling. From studies at single-cell level to animal behavior, recent advances in technology and genetics have revealed the impact of astrocyte activity in brain function from cellular and synaptic physiology, neuronal circuits to behavior. The present review critically discusses the consequences of astrocyte signaling on synapses and networks, as well as its impact on neuronal information processing, showing that some crucial brain functions arise from the coordinated activity of neuron-glia networks.

## Introduction

Brain information processing is conventionally recognized as derived from neuronal activity, with neurons and their dynamic signaling responsible for the transfer and processing of information (Majewska and Sur, 2006). However, the brain also contains other non-neuronal cells, glial cells, which exceed the number of neurons and have been largely ignored as being involved in the processes related with information coding handling by neural networks and underlying brain function. Nonetheless, decisive advances in the characterization of the molecular and physiological properties of astrocytes, a particular type of glial cells have revealed that they may play active roles in neurotransmission and neuronal physiology (Araque et al., 1999; Perea et al., 2009). Accordingly, novel concepts in brain physiology have been coined, such as “tripartite synapse”, to highlight the direct involvement of astrocytes in synaptic function, gliotransmitters, to generically name neuroactive substances released by astrocytes, or gliotransmission, to define the active signaling between astrocytes and neurons (Volterra and Bezzi, 2002).

There is common agreement regarding the crucial roles of astrocytes in controlling the homeostasis of surrounding synapses, with a fundamental role in energy metabolite supply (Hassel et al., 1995; Westergaard et al., 1995; Allaman et al., 2011), clearance of extracellular potassium (Sontheimer, 1994; Kressin et al., 1995; Butt and Kalsi, 2006), and glutamate (Hansson et al., 1985; Bergles et al., 1997; Coulter and Eid, 2012). Moreover, astrocytes enwrapping synapses also control extracellular space volume, and hence the extracellular levels and diffusion of neuroactive substances (Nagelhus and Ottersen, 2013). Besides these homeostatic functions, astrocytes display dynamic signaling with neurons and synapses. They sense neuronal and synaptic activity through activation of ion channels, neurotransmitter transporters and receptors. Activation of these molecules may results in elaborate Ca^2+^ signals into astrocytes. In turn, astrocytes, via uptake or release of gliotransmitters, such as glutamate, ATP, and D-serine, (Santello et al., 2012), can modulate neighboring pre- and postsynaptic neuronal elements inducing functional as well as morphological changes in synapses. However, whether astrocytes are active elements in neural network function and whether and how they play active roles in brain information processing is an open debate (Agulhon et al., 2008; Hamilton and Attwell, 2010; Araque et al., 2014).

In this review, we will present a brief summary of the currently available data that has challenged the classical idea that brain function results exclusively from neuronal activity, suggesting that astrocytes are integral units of neural circuits playing a role in the coding information by neural networks. We will also discuss the available as well as the still required evidence that suggests brain function actually arises from the coherent coordinated activity of Neuron–Glia networks.

## Astrocytes process synaptic information

For a cell to be considered as an active element in the brain coding network, it should be able to: (1) receive incoming information; (2) integrate and code that information; and (3) transfer the information to other cells. Neurons have the ability to perform these actions due to their anatomical characteristics and intrinsic electrical properties. Can astrocytes, which do not display electrical excitability to elicit significant active signals (Sontheimer, 1994), behave as transfer and/or processors of synaptic inputs in a neural network?

While astrocytes lack electrical excitability they show intrinsic cellular excitability based on intracellular Ca^2+^ variations (Charles et al., 1991). More importantly, astrocytes are able to respond to different neurotransmitters that trigger intracellular Ca^2+^ signals (Duffy and MacVicar, 1995; Bezzi et al., 1998; Shelton and Mccarthy, 2000; Araque et al., 2002, 2014). Indeed, astrocytes are able to sense the synaptic activity in different regions induced by several neurotransmitters, such as glutamate, GABA, acetylcholine, ATP or nor-epinephrine (Zorec et al., 2012). A remarkable ability shown by astrocytes is that they sense different neurotransmitters and discriminate between different synaptic inputs. Astrocytes located in stratum oriens of hippocampus differentially sense glutamate released from distinct synapses, i.e., they respond to glutamate released from Schaffer collateral axons of hippocampal CA3 neurons but not to glutamate released from other extrinsic afferences (Perea and Araque, 2005). Likewise, astrocytes located in layer 2/3 barrel cortex selectively respond to the activity of glutamatergic inputs from layer 4 of the same column but not to glutamatergic projections from layer 2/3 of adjacent columns (Schipke et al., 2008), showing preference for sensory incoming inputs rather than lateral cortical signaling. Another example of the response selectivity is provided by astrocytes of the ventrobasal thalamus, which show preferential responsiveness to corticothalamic inputs vs. sensory pathways, in spite that both inputs release the same neurotransmitter glutamate (Parri et al., 2010). Therefore, since astrocytes are able to sense incoming information, and also show selectivity in their responses and discrimination of specific synapse activity, they fulfill the first requirement postulated.

The second requirement postulates that a processor must have the ability to integrate different inputs and elaborate specific responses; that is, perform a nonlinear input–output readout. Do astrocytes display integrative properties of the incoming synaptic information? It has been demonstrated that astrocytes are able to discriminate between the activity of different synaptic inputs releasing different neurotransmitters, i.e., cholinergic and glutamatergic hippocampal synapses belonging to different axon pathways, alveus and Schaffer collaterals pathways, respectively; showing selective Ca^2+^ responses to those inputs (Perea and Araque, 2005). Furthermore, as consequence of simultaneous activity of these synapses astrocyte can integrate these inputs and modulate their Ca^2+^ signal producing a nonlinear input–output response. The astrocyte Ca^2+^ signaling is bidirectionally regulated by synaptic activity, showing an enhancement or depression by low and high synaptic activity, respectively (Perea and Araque, 2005). The integration of different signals also arises in the absence of neural network activity indicating that this modulation is due to cellular intrinsic properties. Thus astrocytes, rather than being simple elements performing a linear readout of synaptic activity are active elements endowed with integrative properties, hence satisfying the second requirement to participate in the information coding by the neuronal networks.

## Brain information processing by neuron–glia networks

The third postulate to consider astrocytes as unit processors in the coding of information by neuronal networks requires that they transfer the information to other elements, i.e., influencing neuronal activity and synaptic transmission. Astrocytes can control the activity of neurons and synapses through the uptake of neurotransmitters or the release of gliotransmitters, thus contributing to neuronal network function.

The ability of astrocytes to release gliotransmitters in response to neuronal activity has been reported in several brain areas (Zorec et al., 2012). Those gliotransmitters activate receptors located at presynaptic and postsynaptic sites in neuronal membranes leading the regulation of neuronal excitability and synaptic transmission and plasticity (Kang et al., 1998; Fellin et al., 2004; Pascual et al., 2005; Perea and Araque, 2005, 2007; Panatier et al., 2006, 2011; Stellwagen and Malenka, 2006; Henneberger et al., 2010; Navarrete and Araque, 2010; Di Castro et al., 2011; Santello et al., 2011; Fossat et al., 2012). These findings indicate that astrocytes signal to neurons, transferring information to other elements in the network, thus satisfying the third postulated requirement.

Moreover, this astrocyte-to-neuron signaling has been revealed to present interesting and sophisticated properties. For example, a single gliotransmitter may exert multiple effects on the neuronal network depending on the receptor subtype and its subcellular membrane location. Indeed, glutamate released by astrocytes increases neuronal excitability by inducing slow inward currents (SICs) in excitatory neurons through activation of postsynaptic NMDA receptors (Parri et al., 2001; Angulo et al., 2004; Fellin et al., 2004; Perea and Araque, 2005; Navarrete and Araque, 2008; Shigetomi et al., 2008; Chen et al., 2012); but it also enhances synaptic transmission through activation of presynaptic metabotropic glutamate receptors (mGluRs) group I (Fiacco and McCarthy, 2004; Perea and Araque, 2007; Navarrete and Araque, 2010; Bonansco et al., 2011; Perea et al., 2014); and stimulates synaptic transmission by activation of presynaptic NMDA receptors in dentate granule cells (Jourdain et al., 2007). In addition, astrocytes release different gliotransmitters with different neuromodulatory effects. The concurrent or differential weight of these effects at individual neurons may strongly affect the degrees of freedom of the system and hence the neuronal output and circuit function. Indeed, considering that a single hippocampal astrocyte has been estimated to contact ~100.000 synapses (Bushong et al., 2002), and that a single gliotransmitter may have different effects depending on the target neurons and neuronal elements (pre- or postsynaptic), as well as the activated receptor subtypes, the variability and complexity of the potential impact of a single astrocyte on neural network operations can be significant.

Additionally, evidence from hippocampal slices has shown that gliotransmission is a regulated process controlled by activation of particular membrane receptors, i.e., protease-activated receptor 1 (PAR-1), but not purinergic G protein-coupled receptors (P2Y1) stimulates astrocytic glutamate release that evokes SICs (Shigetomi et al., 2008), which add further complexity to astrocytic output signaling. The physiological and molecular conditions that control the richness of this signaling, such as the precise release of each gliotransmitter, and the co-release of different gliotransmistters by single astrocytes (Bergersen et al., 2012; Martineau et al., 2013) or by defined astrocyte subpopulations are still unresolved.

Thus, the existence of different gliotransmitters, the different mechanisms of action of a single gliotransmitter, and the cellular selectivity of the effects on particular neurons, such as in the olfactory bulb where astrocytes releasing GABA and glutamate lead to cell-specific modulation of neuronal activity (Kozlov et al., 2006), provide an enormous amount of degrees of freedom to the possible network states.

The above discussed evidence indicates that astrocytes can be considered as information processors in neural networks. These findings may expand the possible functional consequences of a single neurotransmitter in the network; thus, the existence of emergent properties in network activity provided by astrocyte signaling has been recently reported in the hippocampus and cortex. The well-known phenomenon observed in hippocampus called heterosynaptic depression of excitatory synaptic transmission (Lynch et al., 1977) results from the coordinated activity of neurons and astrocytes; that is, the glutamate released by Schaffer collaterals, with excitatory effects in the network, excites postsynaptic CA1 is a well known acronym for that specific region of the hippocampus. To simplify the reading I recommend to keep like it is. pyramidal neurons as well as inhibitory interneurons that in turn activate astrocytes through GABA release. Then, GABA-stimulated astrocytes release ATP that after being degraded to adenosine leads to the synaptic depression of adjacent excitatory synapses (Zhang et al., 2003; Serrano et al., 2006). Another example is provided by the effects of endocannabinoid (eCB) signaling to astrocytes. eCBs are known to retrogradely depress synaptic transmission (Chevaleyre et al., 2006), but they can also trigger intracellular calcium signaling in astrocytes (Navarrete and Araque, 2008). eCB-activation of astrocytes stimulates the release of glutamate, which leads to an heterosynaptic enhancement of excitatory synaptic transmission by activation of mGluRs in the hippocampus (Navarrete and Araque, 2010), and the spike timing-dependent depression by activation of NMDA receptors in neocortex (Min and Nevian, 2012).

## Astrocyte role in cognitive functions, from *in vivo* data to artificial networks

The current knowledge of the astrocyte neuromodulatory roles in neuronal function mainly derives from studies performed in slices, which have great accessibility to explore the particular properties as well as the cellular and molecular mechanisms of neuron-glia signaling. Hence, the impact of astrocytic function on brain activity and animal behavior are still largely undefined. Novel genetic tools have made possible to specifically manipulate astrocyte signaling *in vivo* while maintaining neuronal signaling intact. Recent studies have focused on the role of ATP/Adenosine as well D-serine released by astrocytes (Fossat et al., 2012) in the slow oscillations brain waves, which are related to sleep (Fellin et al., 2009; Halassa et al., 2009). Inhibiting gliotransmission attenuates both the slow cortical oscillations and accumulation of sleep pressure, which caused by prolonged wakefulness periods can impair cognitive function (Yoo et al., 2007); thus, mice with downregulated purinergic gliotransmission do not show cognitive deficits associated with sleep loss (Halassa et al., 2009). It has been recently suggested that one important role of sleep would be the removal of waste products from the brain (Xie et al., 2013). Astrocytes control brain microcirculation (Iadecola and Nedergaard, 2007), and through aquaporin four water channels located in the vascular endfeet facilitate convective flow out of the para-arterial space and into the interstitial space, which is related with removal of waste products made during neuronal activity. The exchange rate of fluids between those spaces is more effective during sleep than during awakening periods, suggesting an important role of astrocytes in sleep function (Xie et al., 2013). In addition, a proper astroglial network signaling is essential for precise synaptic information transferring between neurons as it has been shown in the gap junction proteins connexin 30 (Cx30) and connexin 43 (Cx43) knock out mice, where altered rate of extracellular glutamate and potassium removal during synaptic activity induce impairments in short and long-term synaptic plasticity (Pannasch et al., 2011, 2014), as well as deficits in sensorimotor and spatial memory tasks (Theis et al., 2003; Lutz et al., 2009). Another important role of the astrocyte-neuron signaling relies on the metabolic coupling (Magistretti et al., 1999) and the appropriate energy supports for neuronal activity demand. In this context, cognitive processes and their cellular and molecular substrates (i.e., long-term memory formation), that are high metabolically demanding have been shown to be related with an intercellular trafficking of glucose through astroglial networks (Rouach et al., 2008), and astrocytic lactate transporters (monocarboxylate transporter 4 (MCT4) or MCT1) (Suzuki et al., 2011), where dysfunctions of these lactate transporters causes amnesia and long-term potentiation (LTP) impairments (Suzuki et al., 2011).

Astrocytic role in other critical brain functions such as control of breathing and locomotion have also been reported (Baudoux and Parker, 2008; Gourine et al., 2010), showing that astrocyte–neuron interaction occurs *in vivo* and providing insights into the astrocytic influence to the output of complex neuronal networks and behavior.

Besides the contribution of astrocytes to those homeostatic functions, a challenging question refers to their role in higher brain functions, such as coding information in network activity, and in cognitive processes. Astrocytes immersed in local circuits can sense and respond with Ca^2+^ signals to different sensory stimuli and neurotransmitters; i.e., visual stimuli and somatosensory whisker stimulation (Schummers et al., 2008; Takata et al., 2011; Chen et al., 2012; Navarrete et al., 2012; Perea et al., 2014), indicating that astrocytes are able to perceive incoming sensory inputs. But do they have a role in the coding of sensory information? If they were actively involved in this task, they should locally impact synapses of the sensory system, which would be translated into a modulation of global brain state dynamics. Recent studies focused on the cholinergic system function have revealed the active and crucial roles of astrocytes in information processing by neuronal networks (Takata et al., 2011; Chen et al., 2012; Navarrete et al., 2012). Cholinergic activity is related with changes in brain states, such as during attention and vigilance states (Everitt and Robbins, 1997; Sarter et al., 2005), contributes to hippocampal theta rhythm oscillations (Hasselmo, 2006), and induces synaptic plasticity processes (Picciotto et al., 2012). Thus, these novel studies have shown that astrocytes and gliotransmission are the cellular and molecular mechanisms underlying certain cholinergic effects (Takata et al., 2011; Chen et al., 2012; Navarrete et al., 2012). In the hippocampus, astrocytes respond with Ca^2+^ elevations to cholinergic activity triggered by either sensory stimuli or direct stimulation of cholinergic nuclei and axons (Araque et al., 2002; Perea and Araque, 2005; Navarrete et al., 2012). This astrocyte Ca^2+^ signal is necessary to trigger the synaptic LTP associated with the cholinergic activity through a mechanism that involves the release of the gliotransmitter glutamate and the subsequent activation of mGluRs (Navarrete et al., 2012). In somatosensory and visual cortex, cholinergic activation of astrocytes stimulates the release of D-serine or glutamate, which, in association with whisker (Takata et al., 2011) or visual stimuli (Chen et al., 2012), mediate long-term plasticity changes in cortical sensory responses mediated by NMDA receptors. Notably, the astrocyte-mediated potentiation of visual inputs is indeed stimulus-specific, because only synapses active during cholinergic stimulation display such modulation, indicating that the influence of astrocyte-mediated plasticity is synapse-specific and suggesting the existence of intimate organization of astrocytes and synapses that convey and generate visual-specific responses (Schummers et al., 2008; Chen et al., 2012).

New useful tools have been developed in the last few years to decipher the contribution of specific cellular types to the brain information processing. Specifically, the optical control of cellular activity using optogenetics represents a major technical breakthrough in the field of Neurosciences (Fenno et al., 2011). Exploiting the advantages of optogenetics, astrocytes can be selectively stimulated with light to evaluate their consequences either at the local circuits and global network (Gourine et al., 2010; Sasaki et al., 2012; Chen et al., 2013; Perea et al., 2014; Tang et al., 2014). Astrocytes placed in visual cortex, in addition to sensing visual information, when stimulated with the opsin channelrhodopsin-2 lead to an increase in synaptic transmission by releasing glutamate, which regulates excitatory and inhibitory transmission through activation of presynaptic mGluRs (Perea et al., 2014). Furthermore, astrocytes impact visual responsiveness of both excitatory and inhibitory cortical neurons, showing specific modulation of key neuronal response features (Perea et al., 2014). Thus, astrocytes through the dual control of excitatory and inhibitory drive, influence neuronal integration critical for sensory information processing. As a consequence of differential astrocytic neuronal subtype modulation, changes in the excitatory-inhibitory balance in the local network might impact the final output of the circuit, indicating that astrocytes would be involved in multiple aspects of information coding by cortical networks.

Other accumulating evidence indicates the active role of astrocytes in brain cognitive functions (Parpura et al., 2012). Indeed, behavioral studies have demonstrated the correlation between astrocyte signaling dysfunction and cognitive deficits associated with neurobiological diseases, such as major depressive disorder (Cao et al., 2013; Lima et al., 2014), and Huntington’s disease (Tong et al., 2014). Abnormalities of astrocyte-neuron signaling due to the reduced glial release of ATP in prefrontal cortex, a brain region implicated in attentional processes, decision-making, working memory and processing of emotional stimuli, among others (Goldman-Rakic, 1995), are related with depression-like behaviors (Cao et al., 2013), affecting important functions of this cortical area, such as attentional context, working memory and reversal learning functions (Lima et al., 2014). Furthermore, through glial activation of cannabinoid receptor type 1, astrocytes are shown to be responsible for the impairment in working memory performance induced by marijuana (Han et al., 2012), one of the most common effects of cannabinoid intoxication in humans. Likewise, the dysfunctions in astrocyte-mediated potassium homeostasis in striatal astrocytes (Tong et al., 2014), and AMPA receptors expression Bergmann glial cells (Saab et al., 2012), have strong impact on the fine-tuning control of complex motor behaviors, revealing astrocytes as therapeutic targets for motor phenotype disorders (Tong et al., 2014). Despite these compelling findings, many questions remain open and more effort is still needed to fully understand the molecular mechanisms and the physiological conditions underlying neuron-glia signaling and their relevance for other brain functions.

**Figure 1:**
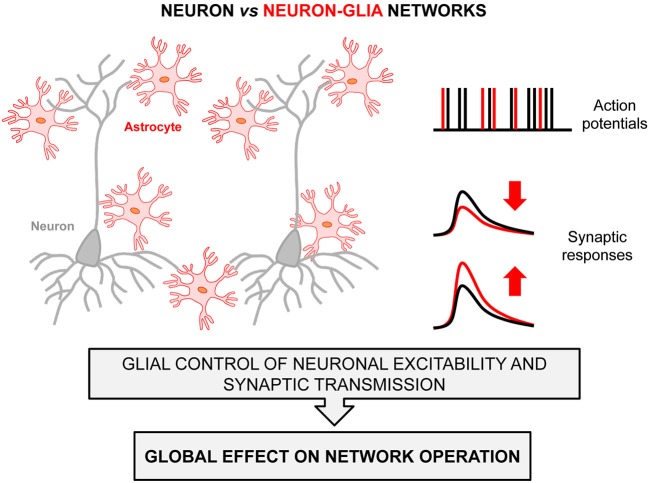
**Neuron-Glia Network scheme. Astrocytes (red) interact with neurons (gray) and impact their cellular excitability (*right-top* panel) and synaptic responses (*right-bottom* panel), which influence important features of information coding by local circuits and hence neural networks operation.** Black and red labels in the right panels denote activity signals of neuron networks versus neuron-glia networks.

Taken together, this growing body of evidence illustrates the existence of Neuron-Glia networks in brain circuits, and demonstrates the ability and influence of astrocytes in neuronal network operations. They show that astrocytes are a structurally and functionally indivisible part of such networks (Figure 1), and reveal how the coordinated signaling pathways established between neurons and astrocytes enables a wide range of, and perhaps all, brain functions.

Finally, in order to fill the gap between the molecular and cellular mechanisms identified by *in situ* studies and *in vivo* behavioral outcomes, the use of mathematical models that include Artificial Neuron-Glia networks appears as a promising approach to better understand the potential emergent properties provided by astrocytes to neural network operation. A pioneering computational study showed that dynamic interaction between astrocytes and presynaptic terminals optimizes synaptic transmission of information (Nadkarni et al., 2008). A more recent study modeling artificial astrocytes in artificial neuronal networks has demonstrated that the presence of astrocytes enhanced the learning potential efficacy of neural networks, i.e., the performance of artificial neuron-glia networks that include artificial astrocytes is improved when compared with similar purely neuronal networks lacking astrocytes (Porto-Pazos et al., 2011). Indeed, including astrocytes into a multilayer feed forward artificial neural network model designed to solve different classification tasks improve the network performance. This relative improvement depends on the intrinsic properties of astrocytes and the strength of the neuron-glia connections and varies with the complexity of the problem tested. Interestingly, the relative efficacy of artificial neuron-glia networks vs. pure neuronal networks increases as the complexity of the network increases (Porto-Pazos et al., 2011), which is in agreement with the gradual increase of the astrocyte-neuron ratio of cells observed in the phylogeny as the nervous system complexity increases. In addition, astrocytes are able to change the threshold value controlling the transition of synchronous to asynchronous behavior among neurons (Amiri et al., 2013). In this way, changes in the interaction properties of astrocyte-neuron signaling lead to the emergence of synchronous/asynchronous patterns in neural responses, showing how astrocytes play a primary role in neuronal firing synchronicity and synaptic coordination (Amiri et al., 2013). Artificial astrocytes through the activation of SICs in neurons participate directly in synaptic plasticity processes, synchronizing postsynaptic activity in neuron clusters and subsequently allow Spike-Timing-Dependent Plasticity based learning to occur at the associated synapses (Wade et al., 2011).

Therefore, the computational evidence of the impact of astrocyte-neuron interactions in neural networks suggests that the richness of biological interactions and brain cognitive functions might emerge from the coordinated activity of Neuron-Glia Networks. Because these computational studies generate in many cases new questions to the field rather than answers, refined future models of Artificial Neuron-Glia Networks implementing more realistic bioinspired network models are needed, which incorporating more features of this complex bidirectional signaling would help to better understand the emergent properties provided by astroytes to network operations. In conclusion, our current knowledge indicates the existence of a rich and complex array of mechanisms underlying astrocyte-neuron interactions that involve different signaling properties determined by multiple elements, e.g., neurotransmitters, gliotransmitters, membrane channels and receptors, intracellular pathways, structural arrangements, etc., and which exert powerful effects on the synaptic function and network operation that underlie brain states and enable behavior.

## Conflict of interest statement

The authors declare that the research was conducted in the absence of any commercial or financial relationships that could be construed as a potential conflict of interest.
